# Does delay in diagnosing colorectal cancer in symptomatic patients affect tumor stage and survival? A population-based observational study

**DOI:** 10.1186/1471-2407-10-332

**Published:** 2010-06-28

**Authors:** Jochim S Terhaar sive Droste, Frank A Oort, René WM van der Hulst, Veerle MH Coupé, Mike E Craanen, Gerrit A Meijer, Linde M Morsink, Otto Visser, Roy LJ van Wanrooij, Chris JJ Mulder

**Affiliations:** 1 Gastroenterology and Hepatology, VU University Medical Centre, Amsterdam, The Netherlands; 2 Gastroenterology and Hepatology, Kennemer Gasthuis, Haarlem, The Netherlands; 3 Epidemiology and Biostatistics, VU University Medical Centre, Amsterdam, The Netherlands; 4 Pathology, VU University Medical Centre, Amsterdam, The Netherlands; 5 Epidemiology and Biostatistics, Comprehensive Cancer Centre Amsterdam, Amsterdam, The Netherlands

## Abstract

**Background:**

Diagnosing colorectal cancer (CRC) at an early stage improves survival. To what extent any delay affects outcome once patients are symptomatic is still unclear.

Our objectives were to evaluate the association between diagnostic delay and survival in symptomatic patients with early stage CRC and late stage CRC.

**Methods:**

Prospective population-based observational study evaluating daily clinical practice in Northern Holland. Diagnostic delay was determined through questionnaire-interviews. Dukes' stage was classified into two groups: early stage (Dukes A or B) and late stage (Dukes C or D) cancer. Patients were followed up for 3.5 years after diagnosis.

**Results:**

In total, 272 patients were available for analysis. Early stage CRC was present in 136 patients while 136 patients had late stage CRC. The mean total diagnostic delay (SE) was 31 (1.5) weeks in all CRC patients. No significant difference was observed in the mean total diagnostic delay in early versus late stage CRC (*p *= 0.27).

In early stage CRC, no difference in survival was observed between patients with total diagnostic delay shorter and longer than the median (Kaplan-Meier, log-rank *p *= 0.93).

In late stage CRC, patients with a diagnostic delay shorter than the median had a shorter survival than patients with a diagnostic delay longer than the median (log-rank *p *= 0.01). In the multivariate Cox regression model with survival as dependent variable and median delay, age, open access endoscopy, number and type of symptoms as independent variables, the odd's ratio for survival in patients with long delay (>median) versus short delay (≤median) was 1.8 (95% confidence interval (CI) 1.1 to 3.0; *p *= 0.01). Tumor-site was not associated with patient survival. When separating late stage CRC in Dukes C and Dukes D tumors, a shorter delay was associated with a shorter survival in Dukes D tumors only and not in Dukes C tumors.

**Conclusion:**

In symptomatic CRC patients, a longer diagnostic and therapeutic delay in routine clinical practice was not associated with an adverse effect on survival. The time to CRC diagnosis and initiation of treatment did not differ between early stage and late stage colorectal cancer.

## Background

Colorectal cancer (CRC) is the second leading cause of cancer-related death in the Western world, with over 4500 deaths per year in The Netherlands and 500,000 deaths per year worldwide [1,2]. Despite advances in surgery and systemic treatment, overall mortality rates have remained relatively unchanged and long-term survival of CRC is 55-58% [1,3,4]. Early diagnosis and prompt treatment are believed to improve survival in patients with CRC. In asymptomatic, average-risk populations, early detection of (pre)malignant lesions by means of biennial faecal occult blood testing (FOBT) has shown to reduce CRC-related mortality [5]. In symptomatic patients, however, the yield of reducing time between onset of symptoms and start of therapy in terms of improved survival is controversial. Conflicting results have been published considering the relationship between the duration of CRC symptoms, tumor stage and survival [6-21]. In the absence of a CRC screening program in the Netherlands, symptoms that could indicate colorectal neoplasia, like rectal bleeding, weight loss and altered bowel habits, form the main indication for colonoscopy. However, symptoms of CRC and its precursors can often be non-specific and diffuse in character while distress of the gastrointestinal tract is common in the general population. Therefore, early recognition of symptomatic CRC can be a challenge for the patient as well as the physician, resulting in a delay in the start of appropriate treatment.

In the present study, we assessed the time between the onset of symptoms and the date of definitive treatment in all patients with symptomatic CRC in a prospective, population-based observational study, evaluating daily clinical practice in the Northern Holland province. In addition to information on delay and disease stage, we analyzed survival of CRC patients after a follow up period of 3.5 years. Our objectives were to evaluate whether a difference would exist in diagnostic delay between symptomatic patients with early stage CRC (Dukes stage A or B) and late stage CRC (Dukes stages C or D), and to correlate survival to duration of diagnostic delay and cancer stage.

## Methods

### Study design

In this multi-centre, population-based, observational study, daily endoscopic clinical practice was prospectively evaluated during a three month period in 2005 in the province Northern Holland [22]. All colonoscopies and sigmoidoscopies performed in this time interval were evaluated. All consecutive patients diagnosed with symptomatic colorectal cancer were registered. The province Northern Holland, serving a total community of 2,599,103 inhabitants in 2005 http://www.cbs.nl, counts 18 hospitals (2 academic hospitals and 16 general/teaching hospitals). All 18 hospitals participated in this study. The study protocol was approved by the central medical ethics review board of the VU University Medical Centre in Amsterdam. When information on diagnostic delay was collected from patient questionnaire-interviews, informed consent was obtained prior to data collection. When information on delay was collected through interviewing the general practitioner or assessing the medical files, no informed consent was needed given the observational nature of this study as judged by the medical ethics review board. Data on survival after 3.5 years of follow up were obtained through the National Cancer Registry up to the 1^st ^of March 2009. For all CRC patients the following items were scored: age, gender, family history of colorectal adenomas and/or cancer, personal history of colorectal adenomas and/or cancer, symptoms attributable to colorectal cancer, symptom duration, mode of presentation, date of first general practitioners visit, date of referral to hospital or endoscopy unit, date of diagnosis, date of onset of treatment or decision to abstain from treatment, endoscopic findings, type of surgery/treatment, surgical findings and histopathology data.

### Study definitions

Diagnostic delay was defined as the time between the onset of the first symptom(s) and the ultimate date of treatment or decision to abstain from further treatment. Total diagnostic delay was divided in patient's delay and healthcare delay. Healthcare delay was subdivided in referral delay, hospital diagnostic delay and staging/treatment delay. The sum of patient's delay and healthcare delay is the total diagnostic delay.

Patient's delay was defined as the time between first onset of complaints and the initial presentation to a doctor for these complaints. Healthcare delay was defined as the time between first doctor's consultation for these complaints and the ultimate date of treatment or until the decision to abstain from further therapy. Referral delay was defined as the time between first doctor's consultation for these complaints and the date of hospital referral. Hospital diagnostic delay was defined as the time between referral date and date of CRC diagnosis. Staging/treatment delay was defined as the time between CRC diagnosis and the ultimate date of treatment or until the decision to abstain from further therapy.

Information on diagnostic delay was collected from patient questionnaire-interviews. If the information could not be collected from the patient, the general practitioner was interviewed. If there were no data available through the general practitioner, the patient's medical files were assessed. If no accurate data on diagnostic delay or tumor stage could be obtained, the patient was excluded. All patient questionnaire-interviews and general practitioner interviews were performed by the first author. Analysis of the patient's medical files was performed by the first author and his co-workers. Data on referral delay, diagnostic delay and staging/treatment delay were collected shortly after the CRC diagnosis within a minimum of 6 months after initiation of treatment.

When comparing diagnostic delay and survival in rectal tumors versus colon tumors, synchronous colon and rectal cancers were excluded. Tumors of the rectosigmoid junction were counted as rectal tumors. Other exclusion criteria were asymptomatic patients (i.e. patients with family history of CRC or screening request) and asymptomatic patients in a surveillance program due to an increased risk for CRC (i.e. post-polypectomy surveillance, post-CRC surveillance, surveillance for hereditary syndromes or surveillance for inflammatory bowel disease). We did include patients in a surveillance program or with a family history of CRC when the colonoscopy was performed for symptoms suggestive of CRC.

CRC was staged according to Dukes using information from the surgical file and/or pathology report [23]. Early stage CRC was defined as Dukes A or Dukes B tumors. Late stage CRC was defined as Dukes C or Dukes D tumors. All cause mortality was used in the survival analyses.

### Data analysis & statistics

We compared patient's delay, healthcare delay and total diagnostic delay in patients with early stage CRC to patients with late stage CRC. We dichotomized the variable delay in total diagnostic delay shorter and longer than the median delay of all patients. The survival of patients with a total diagnostic delay shorter than median was compared to the survival of patients with a total diagnostic delay longer than median by means of Kaplan-Meier analysis. This was done for patients with early stage CRC and late stage CRC separately. In addition to the Kaplan-Meier analysis, we carried out a Cox regression analysis to study confounding and, where necessary, corrected for confounding factors. Age, gender, tumor-site, history of CRC or polyps, synchronous polyps, open-access endoscopy, number and type of symptoms and degree of tumor differentiation were considered as potential clinical and pathological confounders. Kaplan-Meier analysis and Cox regression were also used to study the relation between survival time and tumor stage. Patients lost to follow up were censored at the time last known to be alive. All analyses were performed with SPSS for Windows Version 15 (SPSS Inc., Chicago, Illinois, USA).

## Results

### General results

In total, 376 CRCs were diagnosed after endoscopic evaluation of the large bowel during the three month study period. All CRCs found during emergency abdominal surgery without prior endoscopy (N = 38), were excluded because of insufficient data on diagnostic delay and endoscopic findings. After exclusion of all patients in whom insufficient clinical/histopathological data were collected, the total number of patients available for analysis was 272 (Table 1). In 16% of patients (n = 44), information on patient's delay was collected through patients questionnaire-interviews. In 24% of patients (n = 64), information on patient's delay was collected through interviewing the general practitioners and in 60% of patients (n = 164) this information was collected by assessing the medical files. Within these 272 patients, 136 patients were diagnosed with early stage CRC (Dukes stage A or B) and 136 patients had late stage CRC (Dukes stages C or D). Table 2 shows the details on tumor-site and Dukes classification. The overall mean age (±SD) was 69.5 ± 11 years, range 40-94 years. No differences were observed in mean age between early and late stage CRCs. Forty-nine percent of patients was male (mean age for males 68.2 ± 11 years, mean age for females 70.8 ± 11 years). In 24 patients (9%), CRC was diagnosed in one of the two academic medical centres. The remaining 248 CRCs (91%) were diagnosed in general/teaching hospitals. One hundred patients (37%) had rectal cancer and 167 patients (61%) had colon cancer. In 5 patients (2%) synchronous colon and rectal cancer was found.

**Table 1:** Exclusion criteria and numbers of excluded patients

Family history of CRC (asymptomatic)	3
Post-CRC surveillance program (asymptomatic)	4

Post-polypectomy surveillance program (asymptomatic)	8

Surveillance for hereditary syndromes	3

Surveillance for inflammatory bowel disease	5

Lack of participation of general practitioner	10

Lack of data on patient delay	36

Lack of data on healthcare delay	15

Lack of data on tumor stage	20

Total	104

* In total 376 patients were diagnosed with CRC after endoscopic evaluation of the large bowel during the 3 months study period in this multi-centre, population-based study.

† Abbreviations: CRC = colorectal cancer

**Table 2:** Colorectal cancers (CRC) stratified by tumor-site and Dukes stage in 272 symptomatic patients diagnosed with CRC in a population-based study.

		Dukes classification				
Tumor-site		Dukes A	Dukes B	Dukes C	Dukes D	Total
rectum	N	19	18	18	24	79
	%	24%	23%	23%	30%	100%
rectosigmoid junction	N	3	9	9	5	26
	%	12%	35%	35%	19%	100%
sigmoid colon	N	11	29	12	31	83
	%	13%	35%	15%	37%	100%
descending colon	N	2	3	1	4	10
	%	20%	30%	10%	40%	100%
transverse colon	N	2	7	4	1	14
	%	14%	50%	29%	7%	100%
ascending colon	N	1	16	3	6	26
	%	4%	62%	12%	23%	100%
cecum	N	4	12	7	11	34
	%	12%	35%	21%	32%	100%

Total	N	42	94	54	82	272
	%	15%	35%	20%	30%	100%

In 92% of patients, referral to the hospital followed initial presentation at the general practitioners office and in 8% of patients the initial presentation was on the emergency room which warranted an endoscopic evaluation of the colo-rectum.

In 57% of patients (n = 154), rectal bleeding was the primary indication for endoscopy. In 16% of patients (n = 44) weight loss was the primary indication and a change in bowel habits accounted for 13% (n = 34) of the procedure indications. Twelve percent (n = 32) of patients presented with an iron deficient anaemia and in 3% (n = 8) other indications like abdominal pain, tenesmus or bloatedness were mentioned. Most patients (84%) presented with 2 or more symptoms.

### Diagnostic delay and tumor stage

**Figure 1:**
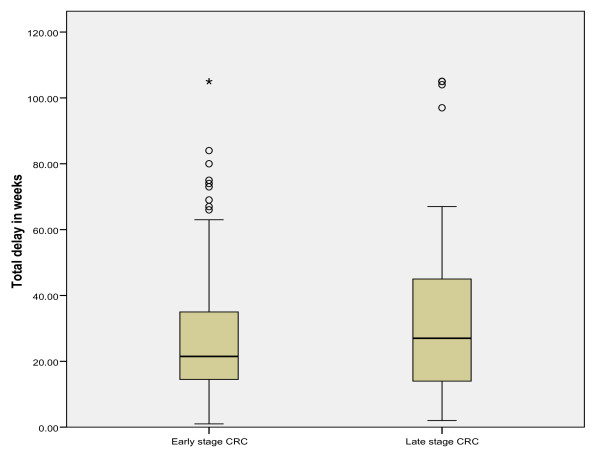
**Mean total diagnostic delay in early and late stage colorectal cancer**.

**Figure 2:**
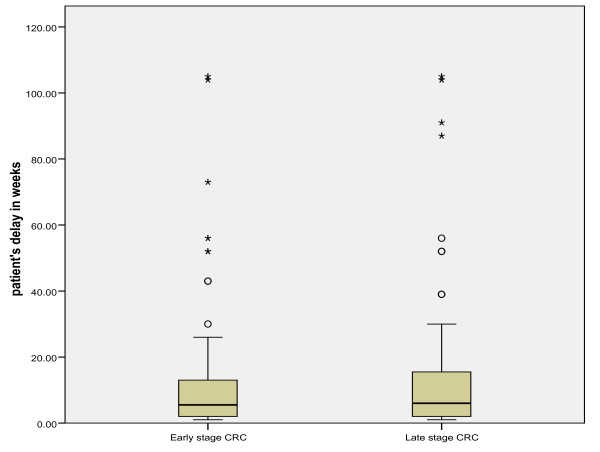
**Mean patient's delay in early and late stage colorectal cancer**.

**Figure 3:**
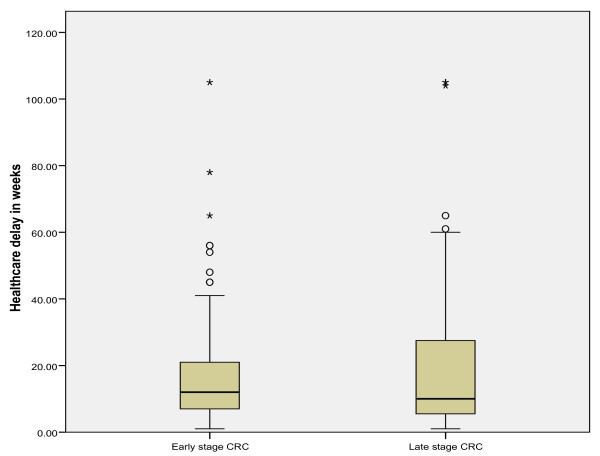
**Mean healthcare delay in early and late stage colorectal cancer**.

The mean total diagnostic delay (SE) was 31.2 (1.5) weeks in all CRC patients (range: within 1 week to >104 weeks). A breakdown of total diagnostic delay into patient's delay and healthcare delay is shown in Table 3, and of healthcare delay into referral delay, hospital diagnostic delay and staging/treatment delay is shown in Table 4. The mean and median diagnostic delay (SD and SE) for early and late stage CRC separately are also shown in Table 3 and 4. Since the data on delay were skewed, we transformed the delay data logarithmically and compared the means using the independent-samples t-test. No significant differences were observed in the mean total diagnostic delay, the mean patient's delay and the mean healthcare delay in early versus late stage CRC (*p *= 0.27*, p *= 0.56 and *p *= 0.46, respectively tested on a logarithmic scale). The absence of differences in diagnostic delay in early and late stage disease is further illustrated by using box plot graphics. Figures 1, 2 and 3 show box plot graphics comparing the total diagnostic delay, patient's delay and healthcare delay in early and late stage colorectal cancer. Even though total healthcare delay did not show significant differences in early versus late stage CRC, specification of healthcare delay in referral delay, hospital diagnostic delay and staging/treatment delay did show that referral delay was significantly longer in late stage CRC compared to early stage CRC (Table 4). Staging/treatment delay, however, was significantly shorter in late stage disease compared to early stage disease.

**Table 3:** Diagnostic delay in early versus late stage colorectal cancer (CRC) in 272 symptomatic patients diagnosed with CRC in a population-based study.

Early vs Late stage CRC		patient's delay (weeks)	healthcare delay (weeks)	total diagnostic delay (weeks)
Early stage CRC	Mean	12.6	17.1	29
(Dukes A&B, N = 136)	Median	5.5	12	21.5
	SD	19.8	15.7	22.9
	SE	1.7	1.3	2
				
Late stage CRC	Mean	15	19	33.4
(Dukes C&D, N = 136)	Median	6	10	27
	SD	23.3	21.4	26.9
	SE	2	1.8	2.3
				
Total	Mean	13.8	18	31.2
(N = 272)	Median	6	12	23.5
	SD	21.6	18.8	25
	SE	1.3	1.1	1.5

**Table 4:** Specification of healthcare delay in early versus late stage colorectal cancer (CRC) in 272 symptomatic patients diagnosed with CRC in a population-based study.

Early vs Late stage CRC		healthcare delay (weeks)*	referral delay (weeks) †	hospital diagnostic delay (weeks) §	staging/treatment delay (weeks) ‡
Early stage CRC	Mean	17.1	6.7	6.1	4.9
(Dukes A&B, N = 136)	Median	12	1	3	4
	SD	15.7	13.9	7.5	3.2
	SE	1.3	1.2	0.6	0.3
					
Late stage CRC	Mean	19	11	5.2	3.6
(Dukes C&D, N = 136)	Median	10	2	2	3
	SD	21.4	20.8	8.2	2.6
	SE	1.8	1.8	0.7	0.2
					
Total	Mean	18	8.8	5.7	4.2
(N = 272)	Median	12	1	3	4
	SD	18.8	17.8	7.9	2.9
	SE	1.1	1.1	0.5	0.2

* No significant difference was observed in the mean healthcare delay in early versus late stage CRC

(p = 0.46; tested on a logarithmic scale using the independent-samples t-test).

† Mean referral delay was significantly longer in late stage CRC compared to early stage CRC

(p = 0.04; tested on a logarithmic scale using the independent-samples t-test).

§ No significant difference was observed in the mean hospital diagnostic delay in early versus late stage CRC

(p = 0.09; tested on a logarithmic scale using the independent-samples t-test).

‡ Mean staging/treatment delay was significantly shorter in late stage CRC compared to early stage CRC

(p < 0.0001; tested on a logarithmic scale using the independent-samples t-test).

**Figure 4:**
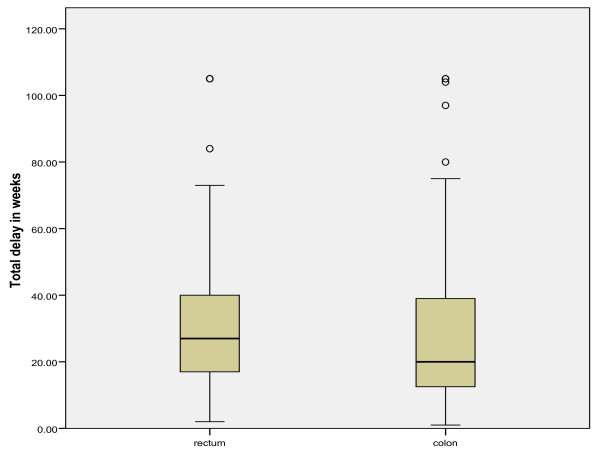
**Mean total diagnostic delay in rectal and colon cancer**.

In colon and rectal cancer separately, the mean total diagnostic delay (SE) was 28.2 (1.7) and 35.4 (2.8) weeks, respectively (*p *= 0.02; tested on a logarithmic scale) (Figure 4). Patient's delay, but not healthcare delay, was the contributing factor for the significantly longer diagnostic delay in rectal cancers as compared to colon cancers (*p *= 0.04 versus *p *= 0.37; tested on a logarithmic scale). No significant differences were observed in diagnostic delay in early versus late stage CRC in either colon or rectal cancers (*p *= 0.98 and *p *= 0.09, respectively).

There were no significant differences in diagnostic delay between the academic centres and the general/teaching hospitals (*p *= 0.44). Obviously, when a patient presented in the hospital at the emergency room, a significantly shorter diagnostic delay was observed as compared to referral via the general practitioner (*p *< 0.0001). No significant differences in tumor stage were found when a patient presented in the hospital via the emergency room as compared to referral via the general practitioner.

### Survival data

**Figure 5:**
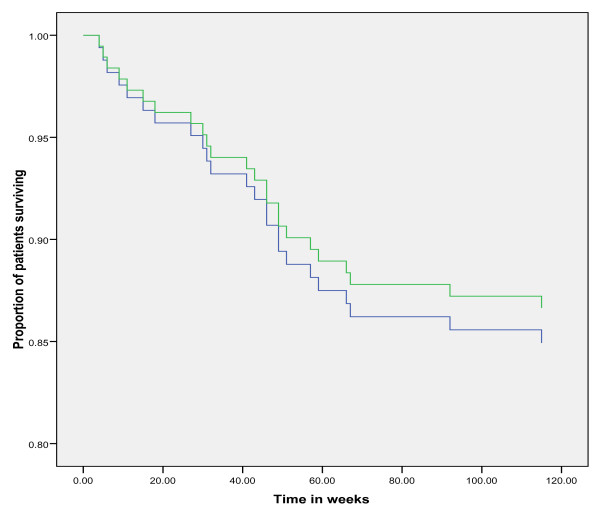
**Survival analysis in early stage colorectal cancer**. Survival in early stage CRC for diagnostic delay longer and shorter than the median delay of 23.5 weeks. On the Y-axis the proportion of patients surviving is plotted. On the X-axis the time is plotted in weeks. Log-rank *p *= 0.93 using Kaplan-Meier analysis. In the Cox regression model with survival as dependent variable and median delay, age, open access endoscopy, number and type of symptoms as independent variables, the odd's ratio for survival in patients with long delay (>median) versus short delay (≤median) was 1.1 (95% confidence interval 0.5 to 2.6; *p *= 0.76). Blue line: delay < median delay. Green line: delay > median delay

**Figure 6:**
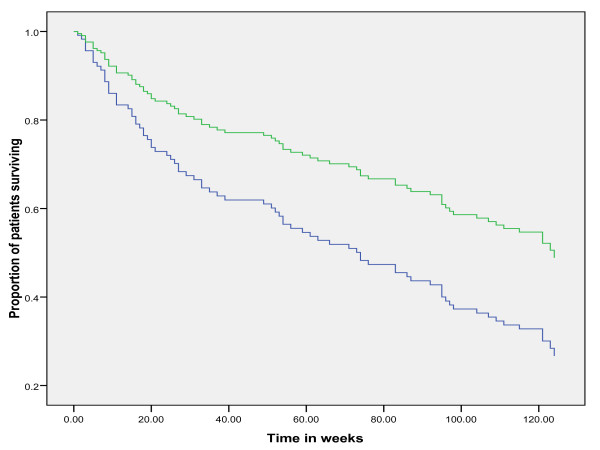
**Survival analysis in late stage colorectal cancer**. Survival in late stage CRC for diagnostic delay longer and shorter than the median delay of 23.5 weeks. On the Y-axis the proportion of patients surviving is plotted. On the X-axis the time is plotted in weeks. Log-rank *p *= 0.01 using Kaplan-Meier analysis. In the multivariate Cox regression model with survival as dependent variable and median delay, age, open access endoscopy, number and type of symptoms as independent variables, the odd's ratio for survival in patients with long delay (>median) versus short delay (≤median) was 1.8 (95% confidence interval (CI) 1.1 to 3.0; *p *= 0.01). Blue line: delay < median delay. Green line: delay > median delay

After 3.5 years of follow up, 41.5% of patients had died, 1.5% of patients were lost to follow up because of emigration and 57% of patients were still alive. Of the 113 patients that had died during the 3.5 years of follow up, 75% had late stage CRC and 25% had early stage CRC. In early stage CRC, no difference in survival was observed between patients with total diagnostic delay shorter versus longer than the median delay (Figure 5, log-rank *p *= 0.93). Correction for potential confounders in a Cox regression analysis did not modify this observation. However, age was independently associated with patient survival. In late stage CRC, patients with a diagnostic delay shorter than the median had a shorter survival than patients with a diagnostic delay longer than the median (Figure 6, log-rank *p *= 0.01). Age and open access endoscopy were independently associated with patient survival in late stage CRC. Also type and number of symptoms were associated with patient survival (rectal bleeding and patients presenting with >2 symptoms were associated with poor prognosis). Tumor-site was not associated with patient survival. In the multivariate Cox regression model with survival as dependent variable and median delay, age, open access endoscopy, number and type of symptoms as independent variables, the odd's ratio (OR) for survival in patients with long delay (>median) versus short delay (≤median) was 1.8 (95% confidence interval (CI) 1.1 to 3.0; *p *= 0.01). When separating late stage CRC in Dukes C and Dukes D tumors, a shorter delay was associated with a shorter survival in Dukes D tumors only and not in Dukes C tumors.

## Discussion

In this prospective, population-based, observational study, we included CRC patients which were diagnosed endoscopically from all hospitals in the Northern Holland province. All symptomatic patients were included, provided that all necessary data on diagnostic delay and tumor stage could be obtained. To date, there is no CRC screening program in place in The Netherlands and no other institutions, like private practices or doctor's offices, are performing endoscopies. Consequently, this study is a reliable representation of symptomatic CRC patients with its diagnostic delay in Northern Holland. Although referral delay was significantly longer in late stage cancers compared to early stage cancers, we observed no significant overall difference in diagnostic delay between early and late stage cancers. In addition, we found no relation between diagnostic delay and survival in early stage cancers. However, in late stage cancers, with Dukes D tumors as the culprit, a shorter delay was associated with poor prognosis. These paradoxical results on the relationship between duration of symptoms, tumor stage and survival, support the idea that the biologic behavior of a given tumor is determined from the very onset, and that this biologic pattern is an important, if not the sole, determinant of the ultimate outcome. It may be surmised that patients presenting after a shorter duration of symptoms may be harboring more virulent and biologically aggressive forms of cancers when compared to patients with symptoms of longer duration. These data may also suggest that, despite increasing tumor cell population, colorectal cancers do not progress in Dukes' stage during the symptomatic period. All colorectal cancers begin as adenomas, according to the Muto and Morson adenoma-carcinoma sequence theory, and it is believed that the progression of these adenomas into cancer takes between 5 and 15 years [24,25]. The symptomatic phase may be a very late event in the natural history of the disease and 1-3 months make little difference in the overall history [26]. This lag time may explain the lack of correlation between diagnostic delay and prognosis. The actual relation between diagnostic delay, tumor stage and survival is complex and might not be elucidated by observational studies. Contradicting results of a recent meta-analysis of observational studies on diagnostic delay and disease stage at diagnosis support this notion [27]. Furthermore, most data regarding delay and survival in CRC patients, suggest that there is no association between diagnostic and therapeutic delay and survival [28].

Surprisingly, we found a distinct longer total diagnostic delay in rectal cancer patients compared to colon cancer patients. Although this finding is consistent with earlier observations, it is somewhat paradoxical that rectal cancer, which has recognizable symptoms, has a longer delay than colon cancer [29]. In the present study, healthcare delay was the major contributor to the delay in diagnosing both rectal and colon cancer. However, patient delay was significantly longer in rectal cancer compared to colon cancer despite the presence of a clear alarming symptom (87% of patients with rectal cancer presented with rectal bleeding, data not shown). It has been described that alarming symptoms do not warrant a doctor's visit and the reasons for this might be unawareness of the importance of symptoms, embarrassment to consult a doctor or fear of a possible cancer diagnosis [30]. In spite of the longer delay in diagnosing rectal cancer, no significant differences were observed in diagnostic delay and tumor stage in both colon and rectal cancers.

The present study carries a number of limitations that need to be considered for proper interpretation of the results. The main limitation is the substantial number of patients with inconclusive delay or histopathology data with its risk of introducing selection bias. Although we defined our study variables beforehand, there was no interventional intent and therefore no standardized reporting format. This means that evaluating daily clinical practice surprisingly showed that not all data on delay were recorded in the medical hospital files or in the general practitioners files.

Furthermore, as in other studies that are based on interview data, the inherent risk of recall bias exists. Although the information on delay was collected shortly after the diagnosis, the often non-specific symptoms of colorectal cancer could not be scored at the time of onset of complaints, increasing the risk of information bias. In this study, we report on robust 3.5 year follow up data. Unfortunately, only all cause mortality was available from the National Cancer Registry, where cancer related mortality data would have been more indicative. Finally, the degree of tumor differentiation, a feature that may reflect the pace of tumor growth, was unaccounted for in 60% of the pathology reports [23]. Therefore, we were not able to reliably correct for the potential confounding effect. However, as the vast majority of CRCs are moderately differentiated, this effect will probably be limited.

## Conclusions

We found no significant difference in diagnostic delay between early and late stage colorectal cancer in symptomatic patients presenting without bowel obstruction. In the present series, a longer diagnostic and therapeutic delay was not associated with worse survival.

## Competing interests

This research project was supported by an unrestricted grant of Nycomed BV, Hoofddorp to "The Amsterdam Gutclub", The Netherlands. This company had no influence on any aspect relevant to this study.

## Authors' contributions

JSTSD was responsible for data acquisition and wrote the paper.

FAO, LMM and RLJW were responsible for data acquisition.

RWMH, MEC and CJJM performed research, devised the study concept and designed research.

OV provided the survival data.

GAM performed critical revision of the article for intellectual content

VMHC performed statistical analysis.

All authors read and approved the final manuscript.

## Pre-publication history

The pre-publication history for this paper can be accessed here:

http://www.biomedcentral.com/1471-2407/10/332/prepub
